# Retraction Note: miR-663 attenuates tumor growth and invasiveness by targeting eEF1A2 in pancreatic cancer

**DOI:** 10.1186/s12943-019-1042-y

**Published:** 2019-07-18

**Authors:** Wenqiao Zang, Yuanyuan Wang, Tao Wang, Yuwen Du, Xiaonan Chen, Min Li, Guoqiang Zhao

**Affiliations:** 1 College of Basic Medical Sciences, Zhengzhou University, Zhengzhou, 450001 Henan Province China; 2 Department of Hemato-Tumor, The First Affiliated Hospital of Henan University of TCM, Zhengzhou, 450000 China

**Retraction note to: Molecular Cancer**

**https://doi.org/10.1186/s12943-015-0315-3**

The Editor-in-Chief has retracted this article [1] because Figure 6b overlaps with Figure 8 of [2] and Figure 4a overlaps with Figure 2b of [3]. An investigation by Zhengzhou University has confirmed that these figures overlap. The data reported in this article are therefore unreliable. Guoqiang Zhao agrees with this retraction. Wenqiao Zang, Yuanyuan Wang, Tao Wang, Yuwen Du, Xiaonan Chen and Min Li have not responded to any correspondence about this retraction.
